# Off-label use of psychotropic drugs in youth

**DOI:** 10.1186/s12888-025-07176-6

**Published:** 2025-07-30

**Authors:** Haakon Gravanti Rosland, Gro Janne Wergeland, Lone Holst

**Affiliations:** 1 Haukeland University Hospital, Bergen, Norway; 2 Department of Child and Adolescent Psychiatry, Division of Psychiatry, Haukeland University Hospital, Bergen, Norway; 3 Department of Clinical Medicine, Faculty of Medicine, University of Bergen, Bergen, Norway; 4 Department of Global Public Health and Primary Care, University of Bergen, Bergen, Norway

**Keywords:** Off-label, Psychotropic medicines, Child and adolescent psychiatry, Psychopharmacology

## Abstract

**Objective:**

Assess the prevalence of off-label psychotropic medication use in paediatric and adolescent outpatients, and to identify the most frequently prescribed drugs for various psychiatric conditions.

**Methods:**

A cross-sectional study was performed using data from the Norwegian Prescription Database (NorPD) and responses to an electronic survey distributed to child and adolescent psychiatrists across Norway. The NorPD data used in this study was collected from January 1, 2019, through December 31, 2019. The electronic survey was sent out in January 2021 and was active until March 2021.

**Results:**

We analysed a total of 55,066 prescriptions, and identified that 55% of these were off-label. When medications for hyperkinetic disorders were excluded, the percentage of off-label prescriptions surged to 95%. Medicines for hyperkinetic disorder had the lowest proportion of off-label use (1%), while the medication class with the most off-label use was other agents, e.g. mood stabilising agents and hypnotics and sedatives (100%). The medicines that were most commonly prescribed were methylphenidate for hyperkinetic disorder, quetiapine for schizophrenia and bipolar disorder, sertraline for depressive disorder and anxiety/obsessive-compulsive disorder.

**Conclusion:**

The off-label usage of psychotropic medications in children and adolescents is prevalent across most psychiatric disorders, with the exception of hyperkinetic disorder. This finding underlines the necessity for ongoing monitoring and assessment of prescription practices, as well as additional research into the safety and efficacy of psychotropic medications in this population.

**Supplementary Information:**

The online version contains supplementary material available at 10.1186/s12888-025-07176-6.

## Significant outcomes

- Off-label use of psychotropics in youth is prevalent and high.
- Medicines for hyper kinetic disorder is the class with least off-label use.
- This is one of two Nordic studies using large registry data to assess prevalence of off-label use of psychotropics in youth and adolescents.

## Limitations

- Variables in the registry data do not account for comorbid illnesses, prescriber speciality or other background information on the patient.
- Dose and duration were not included in the registry data.
- The sample size of the electronic questionnaire was small and may suffer from self selection bias.

## Background

Psychiatric disorders among children and adolescents (hereafter youth unless specified otherwise) are widespread and have been of increasing concern for more than a decade [1]. Globally, psychiatric disorders affect one in seven 10–19 year olds, accounting for almost 15% of the global disease burden of this age group [2]. In Norway, the prevalence of any psychiatric disorders have been found to be almost 7%, with anxiety disorder at 3,84%, depressive disorder at 0,64%, ADHD at 1.54% and disruptive disorder at 1.62% [3]. The psychiatric prevalence is less in Norway than in other countries. For example, in the USA the prevalence for ADHD was 8%, compared to Norway at 1,54% in 2015 [4].

Psychotropic medications, encompassing antidepressants, antipsychotics, antiepileptics, medicines for hyperkinetic disorder, and hypnotics and sedatives, are used for the treatment of these psychiatric disorders. Their prescription frequencies have increased in both youth and adult populations in Europe and the US [5, 6]. Generally, non-pharmacologic treatments are considered the first line of therapy for children and adolescents for most psychiatric disorders [6–9], the most prevalent being cognitive behavioural therapy (CBT), amongst other therapy approaches for different disorders [10]. However, the role of pharmacotherapy remains crucial and should not be dismissed.

Pharmaceutical companies seeking marketing authorisation for a medicine must present it to a governmental body, known as a “medicines agency” [11]. The European Medicines Agency (EU) [12] and the Food and Drug Administration (US) [13] are the two principal western agencies. In the EU, national medicines agencies, such as the Norwegian Medicines Agency, regulate the national market in their respective country [14]. Discrepancies in formal approval between countries may emerge due to varying national medicines agencies providing approval for their respective markets. Only a selected few psychotropic medications have formal approval for paediatric use from medicines agencies in both Europe and the US, with minor differences between the two markets. In 2019, only 14 psychotropic drugs were approved by the Norwegian Medicines Agency for individuals under 18 years old (Table 1). Consequently, the range of medicines clinicians have at their disposal for use in youth is considerably limited.

**Table 1:** On/off-label status for psychotropic medicines in 2019 in Norway

Active pharmaceutical ingredient (API)	Authorised indication	Authorised age
Sertraline	Obsessive-compulsive disorder (OCD)	> 6 years
Fluoxetine	Moderate to severe depressive episode where 4–6 sessions of psychotherapy has yielded no response	> 8 years
Fluvoxamine	Obsessive-compulsive disorder (OCD)	> 8 years
Atomoxetine	Attention deficit hyperactivity disorder (ADHD)	> 6 years
Methylphenidate	Attention deficit hyperactivity disorder (ADHD)	> 6 years
Lisdexamphetamine	Attention deficit hyperactivity disorder (ADHD)	> 6 years
Dexamphetamine	Attention deficit hyperactivity disorder (ADHD)	> 6 years
Guanfacine	Attention deficit hyperactivity disorder (ADHD)	> 6 years
Lurasidone	Schizophrenia	> 13 years
Aripiprazole	Schizophrenia/Moderate to severe bipolar disorder type I	> 15 years/>13 years
Clozapine	Treatment resistant schizophrenia	> 16 years
Haloperidole	Schizophrenia where other pharmacological treatment has failed	> 13 years
Ziprazidone	Acute treatment of bipolar mania	> 10 years
Lithium	Bipolar disorder	-

Clinicians often prescribe medicines outside of governmental approval, termed as ‘off-label,’ due to the limited number of approved medicines for youth. Off-label use is defined as “all intended use outside of the product’s government approval” [15]. Many medicines only have approval for the adult population aged > 18 years; thus, any usage of these medicines in youth is considered off-label. According to a German study on off-label use of psychotropic medicines in adult psychiatry, the authors found a prevalence of approximately 30% [16]. In a Norwegian study on paediatric off-label use, the authors found the prevalence of off-label use to be over 90% in hospitalised patients [17]. The nature of medical approvals often leads to a higher prevalence of off-label use among youth compared to adults. Therefore, assessing the prevalence of off-label use in child and adolescent psychiatry is crucial.

Off-label use is a consequence of the lack of paediatric clinical trials required to secure marketing authorisation for a specific population. Previously, conducting clinical research on children was considered ethically questionable, thereby excluding them from clinical trials [18]. However, particularly after the paediatric regulation of 2007 [19], there has been a paradigm shift. Now, all pharmaceutical manufacturers are required to provide a Paediatric Investigation Plan (PIP) for novel medicines or when applying for new indications. A PIP is a developmental plan ensuring that necessary data is obtained through studies in children to support the medicine’s authorisation for children [20]. In all countries governed by the European Medicines Agency, applications for marketing authorization of new medicines must include study results, both from an adult and paediatric population. These results should align with an agreed Paediatric Investigation Plan (PIP), unless a deferral or waiver exempts them.

Off-label use has been linked to an increased number of adverse events [21], though this remains uncertain [22]. Regardless, off-label use places a heavier burden of responsibility on the prescribing doctor. However, off-label use doesn’t equate to unauthorised use. In many cases, off-label use is not only suitable but necessary and is frequently recommended by therapeutic guidelines. Several guidelines such as those by the National Institute for Health and Care Excellence (NICE) and UpToDate recommend pharmacological therapies, typically after non-pharmacologic strategies have been unsuccessful [6, 8]. The guidelines usually consider medicines with marketing authorisation for the intended use as first line treatment (e.g. fluoxetine for depressive disorder [6] and sertraline for obsessive-compulsive disorder [23]), whereas off-label products usually are considered if therapy with marketing authorisation fails, e.g. sertraline for anxiety disorder [24].

There is a limited amount of research on the prescribing patterns of psychotropics in youth, with no studies conducted in Norway. One study from Denmark, which collected data from patients ages 0–18 years old in a psychiatric outpatient clinic, found that over 95% of the psychotropic medicines, excluding those for treating Attention Deficit Hyperactivity Disorder (ADHD), were prescribed off-label [25].

Norway provides an excellent environment for gathering prescription data due to the Norwegian Prescription Database (NorPD), which collects all prescriptions from Norwegian pharmacies, thereby reflecting all outpatient use nationally [26].

### Aims of the study

Our objectives were threefold:

- To assess the extent of off-label use of psychotropic medicines in child and adolescent psychiatric outpatient settings for youth in Norway and examine off-label prevalence for different classes.
- To identify the types of medicines most frequently prescribed for various psychiatric disorders.
- To examine clinicians’ preferences in choice of treatment for various psychiatric disorders.

We hypothesised that off-label use of psychotropic medicines in youth is high compared to the adult population and is representative to general paediatric medicine.

## Materials and methods

### Study design and data collection

A cross-sectional study was carried out over the period from 1 st January 2019 to 31 st December 2019. Data were collated from the Norwegian Prescription Database (NorPD), supplemented with data from a digital questionnaire.

The NorPD aggregates information from all prescriptions dispensed from Norwegian pharmacies. In Norway, each individual possesses a unique national identity number, which is pseudonymized in the Norwegian Prescription Database (NorPD). This system enables the tracking of medication usage linked to specific, anonymized individuals. The data presented in NorPD show the number of distinct individuals prescribed a particular medication within the study period. For example, if an individual retrieves four boxes of sertraline, at four different times, within one year, it is only counted once. However, due to the limitations of the available data, it is not possible to assess polypharmacy, i.e., the concurrent use of multiple medications by the same individual. It should be noted that NorPD does not capture data regarding in-patient medication use.

A digital questionnaire was developed and disseminated to child and adolescent psychiatrists in Norway, specifically those who were members of the Norwegian Child and Adolescent Psychiatrist Association (NBUPF). Additionally, head managers of various hospital clinics for child and adolescent psychiatry were requested to circulate the questionnaire to their clinical staff.

### Ethical considerations

Prior to commencing the study, an application was submitted to the Regional Committees for Medical and Health Research Ethics (REK). REK deemed approval unnecessary as the study fell outside the purview of healthcare research law. Consequently, the study was carried out without further approval from REK, with endorsement granted by the Norwegian Centre for Research Data (SKIL, formerly NSD as of study application, ref: 584723). The study was registered in the UiBs system for overview and control for research projects named RETTE.

### Pilot study

In order to improve the quality of the questionnaire, a pilot study was conducted with six participants experienced in psychiatry. The formal background of the pilot participants was one pharmacist and five psychiatrists. Based on their feedback, some questions were reworded, and the questionnaire length was reduced to almost half of its original size (89 questions). The final questionnaire, a multiple-choice format with 48 questions, did not require respondents to provide their names. The estimated completion time was approximately 30 min.

### Study population and data collection procedure

Data on psychotropic prescriptions were obtained from NorPD. A formal application was submitted to the Norwegian Institute of Public Health (NIPH) for this purpose. Upon approval from NIPH, the aggregated data was made accessible for analysis. The population was categorised by age (children: 0–12 years, adolescents: 13–18 years) and grouped by geographical healthcare region.

All child and adolescent psychiatrists who were working in Norway and were members of NBUPF were invited to participate in the study. Based on membership data from NBUPF, we estimate that 586 clinicians work in child and adolescent psychiatry. Additionally, follow-up communication was sent to heads of child and adolescent psychiatric clinics in Norway. The follow up was distributed where contact information was available. Informed consent was obtained from all participants.

### Drug and diagnosis classification

Psychotropic medicines were classified using the Anatomical, Therapeutic and Chemical (ATC) classification [27]. Six disorders classified under International Classification of Diseases 10th Revision (ICD-10) were investigated: Schizophrenia, Bipolar Disorder, Depressive Disorder, Anxiety Disorder, Obsessive-Compulsive Disorder, and Hyperkinetic Disorder. ICD-10 codes are internationally recognised diagnostic classifications and are used by The Norwegian Health Economics Administration (HELFO) to reimburse medication costs [28]. The reimbursement codes, directly linked to ICD-10 codes for diseases, were used to couple drug exposure to diagnosis (Table 2).

**Table 2:** ICD-10/ICPC codes for state reimbursements

HELFO codes ICD-10/ICPC	Refund eligible area of use^a^	Diagnosis
-F2/−72	Psychotic or near psychotic symptoms in mental illness requiring treatment.	Schizophrenia
-F3/−73	Mood related disorders in mental illness requiring treatment	Bipolar and depressive disorder
-F4/−74	Treatment requiring anxiety symptoms in mental illness	Anxiety disorder
F90/P81	Hyperkinetic disorder	Hyperkinetic disorder
−70/−70	Significant conduct disorder requiring treatment	Significant conduct disorder requiring treatment

^a^Some uses are not directly reimbursed. For example, antipsychotics for tics/tourettes

### Off-label use definition

A drug was classified as off-label if it was prescribed for use outside its authorised use as defined in the summary of product characteristics (SPC) in the Norwegian national formulary (FK). If a drug was given on an indication without authorised use, or if a drug did not have a reimbursement code in accordance to authorised use, it was deemed off-label. For example, a medicine prescribed for schizophrenia e.g. aripiprazole [29] to a 14-year-old patient was considered off-label, even though it was authorised for 13-year-olds with Bipolar Disorder Type I.

### Statistical analysis

Data analysis was performed using descriptive statistics in IBM SPSS Statistics version 25 [30].

### Descriptive statistics

Demographic and background characteristics of survey participants were summarized using appropriate descriptive statistics. Continuous variables (age, years of experience) were reported with interquartile ranges (IQR) and presented as frequencies (n) and percentages (%). Categorical variables (sex, regional affiliation, specialist status) were presented as frequencies (n) and percentages (%).

### Prevalence calculations

Prevalence of off-label use was calculated as the proportion of off-label prescriptions relative to the total number of prescriptions within each diagnostic category and medication class. Specifically:

- Overall off-label prevalence = (Number of off-label prescriptions/Total number of prescriptions) × 100
- Diagnosis-specific prevalence = (Number of off-label prescriptions for specific diagnosis/Total prescriptions for that diagnosis) × 100
- Medication class-specific prevalence = (Number of off-label prescriptions within medication class/Total prescriptions within that medication class) × 100

Prevalence of psychiatric diagnoses was calculated as the proportion of prescriptions for each diagnostic category relative to the total number of psychotropic prescriptions dispensed during the study period.

### Statistical testing

Chi-squared tests of independence were employed to examine associations between categorical variables, specifically to assess whether prescribing patterns (choice of first-line pharmacological treatment) differed significantly across geographical healthcare regions. The chi-squared test was selected as the appropriate statistical method because both variables (treatment choice and geographical region) were categorical, data consisted of independent observations with mutually exclusive categories and expected cell frequencies met the assumptions for chi-squared analysis (expected frequency ≥ 5 in at least 80% of cells).

The analysis aimed to test independence between two categorical variables rather than compare means or proportions against a known value.

### Statistical significance and reporting

Statistical significance was set at α = 0.05 (*p* < 0.05). For chi-squared tests, results are reported including: degrees of freedom (df), chi-squared test statistic (χ²), critical value at α = 0.05 and exact p-values.

## Results

### Prescriptions data from NorPD

Over the course of the year 2019, a total of 55,066 prescriptions for psychotropic medications were dispensed for children and adolescents (0–18 years old) in Norway. Notably, 55% of prescriptions were identified as off-label regarding indication and age. Upon excluding medications for hyperkinetic disorder (HKD), the off-label usage prevalence increased to 95%. Off-label frequencies were distributed unequally per medication class (Table 3). The most common diagnosis was HKD with 22,257 prescriptions, while schizophrenia (SHZ) was the least common with 838 prescriptions (Table 4).

**Table 3:** Off-label frequency per drug class (*N* = 55 066)

Drug class	ATC- classification	Number of off-label prescriptions	Number of on-label prescriptions	Total (*n*)	Off-label frequency (%)
Antidepressants	N06A	4 166	1 444	5 610	64
Antipsychotics	N05A	2 304	1 296	3 600	64
Agents for hyperkinetic disorders	N06B + C02A C	225	22 233	22 458	1
Other agents, incl. antiepileptic agents with mood stabilising effect and hypnotics and sedatives^a^	N03A + N05C	23 398	0	23 398	100

^a^Each of the included drug classes demonstrates a 100% off-label frequency, meaning that none of these medications are officially authorised for the indications for which they were prescribed and were therefore grouped together

**Table 4:** Number of prescriptions per indication (55 066)

Indication	Number of prescriptions, *n* (%)
Schizophrenia	838 (1.5%)
Depression	3 347 (6.1%)
Bipolar Disorder	1 767 (3.2%)
Anxiety Disorder/Obsessive Compulsive Disorder	1 798 (3.3%)
Hyperkinetic Disorder	22 257^b^ (40.4%)
Other (Unclassified)^a^	25 059 (45.5%)

^a^E.g. Antiepileptic medicines, hypnotics and sedatives etc

^b^Number of prescriptions for HKD differs in Table 4 from total numbers of agents for HKD in Table 3 by 201. This discrepancy is because Table 3 show total number within the drug class, both with and without diagnosis. Table 4 shows only total prescriptions for patients with a HKD diagnosis

### Prescribing patterns across diagnostic categories

#### Schizophrenia

Among children younger than 13 years, the prescription of antipsychotics was limited (*n* = 34), with aripiprazole (56%) and risperidone (44%) being the only prescribed medicines. For adolescents (13–18 years old) diagnosed with SHZ, quetiapine (34%) was most frequently prescribed, followed by aripiprazole (32%), olanzapine (19%), and risperidone (12%).

#### Bipolar disorder

In the group of children younger than 13 years (*n* = 291), almost half of the prescribed drugs were antiepileptic drugs with mood stabilising effects, the rest being Second Generation Antipsychotics (SGAs). The antiepileptic medicines prescribed were lamotrigine (27%) and valproate (21%), while the SGAs included quetiapine (31%), aripiprazole (16%), and risperidone (5%). Among adolescents (13–18 years old) diagnosed with Bipolar Disorder (BPD), quetiapine was the most commonly prescribed drug, accounting for 50% of prescriptions. Remaining medicines were lamotrigine (18%), aripiprazole (13%), olanzapine (8%), risperidone (7%) and valproate (4%).

#### Depressive disorder

In the group of children younger than 13 years (*n* = 76), sertraline was most commonly prescribed (93%), followed by fluoxetine (7%). Among adolescents aged 13–18 years diagnosed with Depressive Disorder (DD), sertraline (39%) was the most frequently prescribed drug followed by fluoxetine (23%), escitalopram (20%), mirtazapine (9%), venlafaxine (3%) and mianserine (2%).

#### Anxiety and obsessive-compulsive disorder

In the group of children younger than 13 years (*n* = 153), sertraline was most commonly prescribed (94%), followed by fluoxetine (6%). Sertraline was also the most frequently prescribed drug for the treatment of adolescents diagnosed with Anxiety Disorder (AD) and/or Obsessive-Compulsive Disorder (OCD) with 68%, followed by escitalopram (16%) and fluoxetine (13%).

#### Hyperkinetic disorder

In the group of children younger than 13 years (*n* = 10 126), methylphenidate was most commonly prescribed (68%), followed by lisdexamphetamine (18%), then atomoxetine (9%) and guanfacine (3%). Dexamphetamine was least used with 2% of all prescriptions.

In the group of adolescents (*n* = 12 131), the pattern was similar with methylphenidate as the most commonly prescribed (71%), followed by lisdexamphetamine (18%), then atomoxetine (8%) and guanfacine (2%). Dexamphetamine only accounted for 1% of prescriptions.

### Survey responses from clinicians in child and adolescent psychiatry

A total of 117 child- and adolescent psychiatrists initiated the survey, of which 68 completed it (58% completion rate). Considering the 586 clinicians who received the survey, the actual response rate was 12%. The respondents represented all geographical regions of Norway, with varied experience in child and adolescent psychiatry (Table 5).

**Table 5:** Demographic and background information of survey participants (medical Doctors working in child and adolescent psychiatry)

		Total participants = 68 (*n*)	Percentage (%)
**Sex**	Male	19	28
	Female	48	71
	No information	1	1
**Age (years)**	20–39	16	4
	40–49	25	37
	50–59	15	22
	60+	12	18
**Experience as a licensed specialist in child and adolescent psychiatry (years)**	Not a specialist	17	25
	0–5	14	19
	6–10	13	19
	11–20	15	22
	20+	9	13
**Regional affiliation (Norway Regional Health Authority)**	South-Eastern^a^	29	44
	Western	15	21
	Central	9	13
	North	13	19
	No information	2	3

^a^The South-Eastern region includes major cities including the capital Oslo

### Off-label choices as first line treatment

**Fig. 1:**
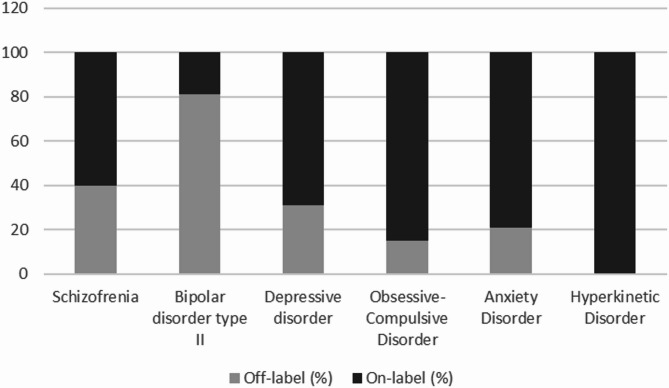
Off-label choices as first line treatment for different diagnostic classifications

A notable 45% of the clinicians suggested an off-label drug as the first-line treatment for a psychiatric disorder. The off-label choices varied across different diagnoses (Fig. 1).

### Prescribing patterns

#### First line pharmacological treatment

For SHZ, the majority of respondents (60%) chose aripiprazole as the first line treatment. For BPD Type I, there was no clear preference, while for BPD Type II, lamotrigine was the most preferred medicine (44%). For DD, fluoxetine was most preferred (69%). For AD and OCD, sertraline was the most preferred treatment (respectively 79% and 85%). Lastly, for HKD, methylphenidate slow release was the first line treatment of choice for 89% of respondents.

#### Second and third line pharmacological treatment

The preferred second and third line treatments for HKD were lisdexamfetamine (60% for second line) and atomoxetine (53% for third line). For other mental disorders, there was no clear preference for second- and third-line treatment options.

### Prescribing differences between geographical regions

While no significant differences in first line treatment choices were observed across geographical healthcare regions (four in total), a chi-squared test revealed statistically significant differences in prescription patterns between these regions (Table 6). All *p*-values were less than 0,05, indicating that it is highly unlikely that the results are there by chance.

**Table 6:** Differences in preferred choice of first line treatment by geographic region using Chi-Square

Disorder	Degrees of freedom	Chi-square	Critical value	*P*-value
Schizophrenia	12	38.2	21.0	< 0.001
Bipolar disorders	9	42.8	16.9	< 0.001
Depressive disorder	18	60.3	28.9	< 0.001
Obsessive-compulsive and anxiety disorders	6	13.2	12.6	0.0399
Hyperkinetic disorder	15	131.2	25.0	< 0.001

## Discussion

### General

The principal objective of the study was to investigate the frequency and patterns of off-label psychotropic drug use in children and adolescents within Norway for the year 2019. The study identified a significant prevalence of off-label psychotropic prescriptions, with the percentage escalating to 95% when prescriptions for HKD were omitted. These findings align with our initial hypothesis and correlate with previous investigations. For instance, Nielsen et al. discovered similar outcomes in their Danish study examining off-label psychotropic use, with an over 95% off-label prevalence when HKD prescriptions were excluded [22]. This level of similarity is expected given the commonalities in healthcare structures and marketing authorisations for medications between the Nordic countries [31]. As hypothesised, the off-label use of psychotropic medications in youth exceeds that in adult psychiatry, aligning with trends observed in general paediatric medicine. This pattern is a natural consequence of the processes governing approvals of medications.

### Specific medication classes

#### Medicines for hyperkinetic disorder

In the context of specific medication classes, the lowest off-label prescription prevalence was observed in the case of medicines for HKD, and HKD prescriptions constituted almost half of all psychotropic prescriptions for the youth demographic. This indicates a preference among practitioners to initiate pharmacologic treatments for disorders where more research has been undertaken regarding efficacy, safety, and treatment hierarchy than for other psychiatric disorders for youth. This practice aligns with the national guidelines issued by the Norwegian Health Directorate (Hdir), which are formulated on the basis of the NICE-guidelines [32, 33].

#### Antidepressants

A noticeable discrepancy was found between the high retrieval frequency of sertraline and escitalopram for DD and the clinical preference for fluoxetine, the only authorised product for DD in individuals over 8 years old [34]. This incongruity might be attributed to the diverse study population represented in the questionnaire and NorPD. The survey only included doctors working in child and adolescent psychiatry, while the prescribers reflected in the NorPD data could be from any medical specialty. However, considering all pharmacological treatments for mental health conditions, according to the Norwegian Directorate of Health, should be initiated by a child and adolescent psychiatrist [35], the NorPD data somewhat reflect prescribing patterns of this specialist group.

We could not separate AD from OCD in our dataset, due to how we deem off-label prevalence. Therefore, all prescriptions with reimbursement code F4 were considered on-label if the medication was approved for either OCD or AD. In Norway, only OCD has authorization for the paediatric population, meaning all pharmacologic treatment of AD is off-label. Our data will therefore underestimate off-label use of antidepressants for these psychiatric diagnoses.

### Antipsychotics

#### Mood-stabilizing agents, Hypnotics and Sedatives

For mood-stabilizing agents, hypnotics and sedatives, all prescriptions were off-label since none of the medicines had marketing authorization for the paediatric population for psychiatric diagnoses. The most frequently prescribed medication here was melatonin. For the study period, melatonin did not have marketing authorization, and was therefore deemed off-label.

#### Statistical significance and geographical differences

Statistically significant differences in choice of pharmacologic treatment across different geographical healthcare regions were found in the NorPD data, which was surprising given the consensus among regions reported in the questionnaire. Tarjei Widding‑Havneraas et al. found in 2022 that the variation of diagnosis of ADHD was larger than what can be explained by geographical variations in symptom levels [36]. The level of diagnosing psychiatric illnesses will of course influence the frequency of prescription. Further research with more controlled variables is warranted to examine the variations in prescribing practices between geographical regions.

### Strengths and limitations

These study findings need to be interpreted in light of some limitations. The use of a national register of medication purchases from pharmacies in Norway and linking the product to its intended use via national reimbursement codes may not completely capture off-label usage. Due to distribution challenges, our electronic survey was unable to encompass all medical practitioners who prescribe psychopharmacologic treatment. Given that national guidelines advocate for the initiation of psychopharmacologic treatment in specialist care, we believe that the responses obtained from the survey effectively illustrate prevailing prescribing practices. There was also no way of examining polypharmacy with the provided data, meaning that the same individuals could account for several medicines. The diagnoses represented by reimbursement codes are clinician-reported, making them subjective. Different clinicians may apply varying cut-offs, potentially leading to discrepancies in off-label use. In addition, the NorPD does not differentiate among prescribers, adding a level of uncertainty as to whether the prescriber is a specialist in child and adolescent psychiatry or another field of medicine. Also, the low response rate to the questionnaire compared to the total number of clinicians surveyed could result in self-selection bias.

### Clinical perspectives

From a clinical perspective, this study should enhance awareness among prescribers about the limited evidence base supporting the use of psychotropic medications for many conditions in children and adolescents [37], and the responsibility they bear when prescribing off-label. Clinicians can use the study findings to compare and reflect on their own practices. Off-label prescribing is not inherently problematic, and is often necessary. We advocate for additional research to thoroughly assess the risks and benefits of off-label prescribing. This will help to determine the extent to which this practice poses problems and to illuminate the implications of such trends more clearly.

## Conclusion

In conclusion, this study underlines the extensive off-label use of psychotropic medicines among children and adolescents in Norway, revealing an urgent need for further research and a deeper understanding of these medications within the paediatric population. The authors strongly advocate for researchers, regulatory authorities, and the pharmaceutical industry to concentrate efforts on generating robust safety and efficacy data for psychotropic drugs in this demographic group.

## Supplementary Information

Supplementary Material 1.

## Data Availability

The data that support the findings of this study are available from the first author, HGR, upon reasonable request.

## Abbreviations

CBT: Cognitive behavioural therapy
PIP: Paediatric Investigation Plan
NICE: National Institute for Health and Care Excellence
ADHD: Attention Deficit Hyperactivity Disorder
NorPD: Norwegian Prescription Database
REK: Regional Committees for Medical and Health Research Ethics
SKIL (formerly NSD): Norwegian Centre for Research Data
NBUPF: Norwegian Child and Adolescent Psychiatrist Association
NIPH: Norwegian Institute of Public Health
ATC: Anatomical, Therapeutic and Chemical
HELFO: The Norwegian Health Economics Administration
SPC: Summary of product characteristics
FK: Felleskatalogen
ICD-10: International Classification of Diseases 10th Revision
HKD: Hyperkinetic disorder
SHZ: Schizophrenia
SGAs: Second Generation Antipsychotics
BPD: Bipolar Disorder
DD: Depressive Disorder
AD: Anxiety Disorder
OCD: Obsessive-Compulsive Disorder
