# Supplementary material for: Off-label use of psychotropic drugs in youth
Source: BMC Psychiatry. 2025 Jul 30;25:739. doi: 10.1186/s12888-025-07176-6 (PMC12312274; doi:10.1186/s12888-025-07176-6)
Supplement: Supplementary file 1 — Supplementary Material 1. [file 12888_2025_7176_MOESM1_ESM.docx]

# Supplementary tables

**Table S1**. Off-label prevalence for each psychotropic compound prescribed in Norway in 2019

| **Active pharmaceutical ingredient** | **ATC-code** | **Off-label (%)** |
| --- | --- | --- |
| *Klonazepam* | N03AE01 | 100 |
| *Topiramat* | N03AX11 | 100 |
| *Zopiklon* | N05CF01 | 100 |
| *Zolpidem* | N05CF02 | 100 |
| *Melatonin** | N05CH01 | 100 |
| *Modafinil* | N06BA07 | 100 |
| *Prochlorperazine* | N05AB04 | 100 |
| *Haloperidole* | N05AD01 | 100 |
| *Lurasidone* | N05AE05 | 0 |
| *Amisulpride* | N05AL05 | 100 |
| *Olanzapine* | N05AH03 | 100 |
| *Quetiapine* | N05AH04 | 100 |
| *Aripiprazole* | N05AX12 | 10 |
| *Ziprazidone* | N05AE04 | 67 |
| *Risperidone* | N05AX08 | 22 |
| *Flupenthixole* | N05AF01 | 100 |
| *Zuklopenthixole* | N05AF05 | 100 |
| *Chlorprothixen* | N05AF03 | 100 |
| *Levomephromazine* | N05AA02 | 100 |
| *Perphenazine* | N05AF03 | 100 |
| *Valproate* | N03AG01 | 100 |
| *Lamotrigine* | N03AX09 | 100 |
| *Amitryptiline* | N06AA09 | 100 |
| *Mirtazapine* | N06AX11 | 100 |
| *Mianserine* | N06AX03 | 100 |
| *Bupropione* | N06AX12 | 100 |
| *Nortriptiline* | N06AA10 | 100 |
| *Duloxetine* | N06AX21 | 100 |
| *Trimipramine* | N06AA06 | 100 |
| *Vortioxetine* | N06AX26 | 100 |
| *Citalopram* | N06AB04 | 100 |
| *Paroxetine* | N06AB05 | 100 |
| *Sertraline* | N06AB06 | 46 |
| *Escitalopram* | N06AB10 | 100 |
| *Venlafaxine* | N06AX16 | 100 |
| *Fluoxetine* | N06AB03 | 30 |
| *Guanfacine* | C02AC02 | 9 |
| *Dexamphetamine* | N06BA02 | 3 |
| *Methylphenidate* | N06BA04 | 1 |
| *Lisdexamphetamine* | N06BA12 | 0 |
| *Atomoxetine* | N06BA09 | 1 |

*Melatonin was during the study period unregistered, and therefore off-label. Melatonin has later received marketing authorization for the paediatric population.
